# Prevalence of lower urinary tract infection in South Indian type 2 diabetic subjects

**DOI:** 10.4103/0971-4065.57107

**Published:** 2009-07

**Authors:** J. Janifer, S. Geethalakshmi, K. Satyavani, V. Viswanathan

**Affiliations:** Department of Microbiology, M.V. Hospital for Diabetes and Diabetes Research Centre, [WHO Collaborating Centre for Research Education and Training in Diabetes], Chennai - 600 013, India

**Keywords:** Antimicrobial pattern, causative pathogens, prevalence, type 2 diabetes, lower urinary tract infection

## Abstract

This study was done to determine the prevalence of lower urinary tract infection (UTI), the causative pathogens, their antimicrobial pattern, and the recurrence of infection in type 2 diabetic subjects. A total of 1157 (M: F 428: 729) type 2 diabetic subjects were selected for this study. Midstream urine specimens were collected and the culture tests were done by a quantitative method whereas antimicrobial sensitivity was determined by using the Kirby-Bauer method. A significant colony count was seen in 495 (42.8%) subjects and an insignificant count in 350 (30.3%) subjects; there were a few cases of recurrent UTI. Women (47.9%) had a significantly higher prevalence of UTI than men (34.1%) (χ^2^ = 20.3, *P* < 0.0001). Except for BMI, UTI was significantly associated with age, duration of diabetes, and poor glycemic control in both sexes. About 533 pathogens of gram positive and gram negative bacilli were isolated from 495 subjects in this study. *Escherichea coli (E. coli)* was the most commonly found organism. Gram negative pathogens were found to be highly sensitive to sulbactum / cefoperazone and piperacillin / tazobactum. The prevalence of UTI was significantly higher in women than men with *E. coli* being the major isolated pathogen. Gram negative pathogens were highly sensitive to sulbactum / cefoperazone and piperacillin / tazobactum.

## Introduction

An association between urinary tract infection (UTI) and diabetes mellitus was noted in an autopsy series reported in the 1940s.[1] The urinary tract is the principal site of infection in diabetes. Changes in host defence mechanisms, the presence of diabetic cystopathy and of microvascular disease in the kidneys may play a role in the higher incidence of UTI in diabetic patients.[2] Urinary tract infections are the most commonly found bacterial infections, accounting for nearly seven million office visits and one million emergency department visits, resulting in 100,000 hospitalizations of women, the elderly, and patients with spinal cord injuries and/or catheters, multiple sclerosis, HIV, and also diabetes.[3]

Several severe and less commonly encountered UTIs are thought to occur more frequently in diabetic patients.[4] In a recent study from Europe, asymptomatic bacteriuria was more prevalent among women with diabetes (26%) than in women without diabetes (6%).[5] Diabetic patients are at a high risk of development of UTIs, so it is recommended that special attention is paid to them, especially for the management of bacterial UTIs.[6] Various risk factors such as sexual intercourse, age, duration of diabetes, glycemic control, and complications of diabetes are associated with UTI.[7]

Antimicrobial therapy should be guided both by *in vitro* sensitivity and clinical response. Asymptomatic bacteriuria in excess of 100,000 microorganisms/mL is also an indication for treatment. In either case, a 7–10 day course of the appropriate drug should eradicate the infection although this must be confirmed by re-culture.[8]

The aims of this study were to determine the prevalence of lower urinary tract infection, the causative pathogens, their antimicrobial pattern, and the recurrence in type 2 diabetic subjects.

## Materials and Methods

A total of 1157 (M: F 428: 729) consecutive type 2 diabetic subjects were studied during a period of one year. Demography, anthropometry, and the duration of diabetes were recorded and the body mass index (BMI) (kg/m^2^) was calculated using height and weight measurements. Diagnosis of diabetes was made based on the WHO criteria.[9] Subjects who received antimicrobial drugs during the past one month, pregnant women, and those with involvement of upper tract and renal failure were excluded from the study. The Ethics committee of the institution approved the study and written informed consent was obtained from all the study subjects.

Midstream urine samples were collected from the patients after giving proper guidelines. The urine samples were immediately transported to the microbiology department. If the urine specimen was found to be contaminated with normal flora of the vagina and urethra, the subject was asked to submit another sample for analysis.

Samples were processed using the following standard microbiological procedures: Smears for Gram's staining,[10] culture for morphology, biochemical tests for identifying the species of the pathogens, and antimicrobial sensitivity by the Kirby-Bauer Method.[11] Quality control procedures were incorporated to assure the quality of the stains, media, biochemicals, and antibiotic discs.

A diagnosis of UTI was made if the urine cultures had >10^3^ to >10^5^ colony forming units (CFUs)/mL of a single potential pathogen or two potential pathogens. A pure culture of *Staphylococcus aureus* was considered to be significant regardless of the number of CFUs. The presence of yeast in any number was also considered to be significant.[12]

Glycosylated hemoglobin (HbA_1C_%) was estimated by an immunoturbidimetric method using the Hitachi 917 autoanalyzer. The body mass index and HbA_1C_% were available for a subsample (n = 400).

### Statistical analysis

Data were expressed as mean, SD, and percentages. Student's ‘*t*’ test, Z test, and Chi-square test were used as required; *P* < 0.05 was considered significant. Analysis was performed using statistical package SPSS version 10.0 (SPSS, USA).

## Results

Four hundred ninety-five (42.8%) subjects showed a significant colony count whereas 350 (30.3%) subjects had an insignificant colony count. No growth was seen in 281 (24.3%) specimens; there were 31 (2.7%) improperly collected specimens (if the colony count was >10^5^ CFU/mL with three different organisms). Polymicrobial urinary tract infections were seen in 34 (2.9%) cases and 533 pathogens were isolated among the patients who had UTI. Symptomatic UTI was noted in 298 (60%) subjects.

Table 1 shows the genderwise prevalence of urinary tract infection. Women (47.9%) had a significantly higher prevalence of urinary tract infection than men (34.1%) (χ^2^ = 20.3, *P* < 0.0001). A total of 108 (21.8%) subjects were treated with oral hypoglycemic agents, 172 (34.7%) with insulin while the rest required combination therapy. The HbAC of those with and without were 9.5±2.2 and 8.1±1.9 respectively (*P*<0.0001).

**Table 1:** Genderwise prevalence of urinary tract infection

	*N*	*%*	Men *vs* women Chi square, *P* value
Total (*n* = 1157)	495	42.8	
Men (*n* = 428)	146	34.1	20.3, < 0.0001
Women (*n* = 729)	349	47.9	

Table 2 shows the clinical characteristics of patients with UTI. The prevalence of UTI was not very marked until the age of 45 years and thereafter, a significant increase was seen in both men and women. We found that the rates of the incidence of UTI increased with the increasing duration of diabetes.

**Table 2:** Clinical characteristics of subjects with significant urinary tract infection

Clinical characteristics	Men *n* = 146	Women *n* = 349
Age (years)		
<45 (*n* = 46)	12 (8.2)	34 (9.7)
45–55 (*n* = 137)	35 (23.9)	102 (29.2)
>55 (*n* = 312)	99 (67.8)	213 (61)
	χ^2^ = 116.6,	χ^2^ = 206.6,
	*P* < 0.0001	*P* < 0.0001
Duration of diabetes (years)		
<10 (*n* = 209)	61 (41.8)	148 (42.4)
≥10 (*n*= 286)	85 (58.2)	201 (57.6)
	Z = 1.8,	Z = 2.69,
	*P*= 0.07	*P*= 0.007
BMI (kg/m^2^)*	(*n* = 114)	(*n* = 286)
<25 (*n* = 152)	59 (51.7)	93 (32.5)
≥25 (*n* = 248)	55 (48.2)	193 (67.5)
	Z = 0.18,	Z = 5.5,
	*P* = 0.85	*P* < 0.001
HbA_1c_(%)		
<8 (*n* = 85)	22 (19.3)	63 (22)
8–9 (*n* = 64)	15 (13.2)	49 (17.1)
≥9 (*n* = 251)	74 (64.9)	177 (61.9)
	χ^2^ = 54.1,	χ^2^ = 101.7,
	*P* < 0.0001	*P* < 0.0001

* Men vs Women χ^2^ = 11.99; *P* < 0.0005; Values are *n* (%)

The percentage of patients with UTI varied among the nonobese (BMI < 25 kg/m^2^) and obese (BMI ≥ 25 kg/m^2^) women (Z = 5.5, *P* < 0.001) whereas it did not differ in men. Significant differences were noted between men and women. (Chi^2^ = 11.9, *P* = 0.0005). Poor glycemic control was significantly associated with UTI in both sexes. Age, duration of diabetes, and glycemic control did not show any significant differences between men and women.

About 533 pathogens were isolated from 495 subjects with UTI, out of which, 362 were gram negative bacilli, 100 were gram positive cocci, and 71 were of the *Candida* spp.

Figure 1, panel a shows the percentage-wise distribution of gram negative bacilli in which 258 (71.3%) of the patients had *E. coli*, 49 patients (13.5%) had *Klebsiella* spp., and 32 patients (8.8%) had *Pseudomonas* spp. *Enterobacter* spp. and *Citrobacter* spp. were present in only 2% of the gram negative bacilli-infected cases. *Nonfermenting gram negative bacilli* and *Proteus spp.* both were found only in 1% of the patients.

**Figure 1:**
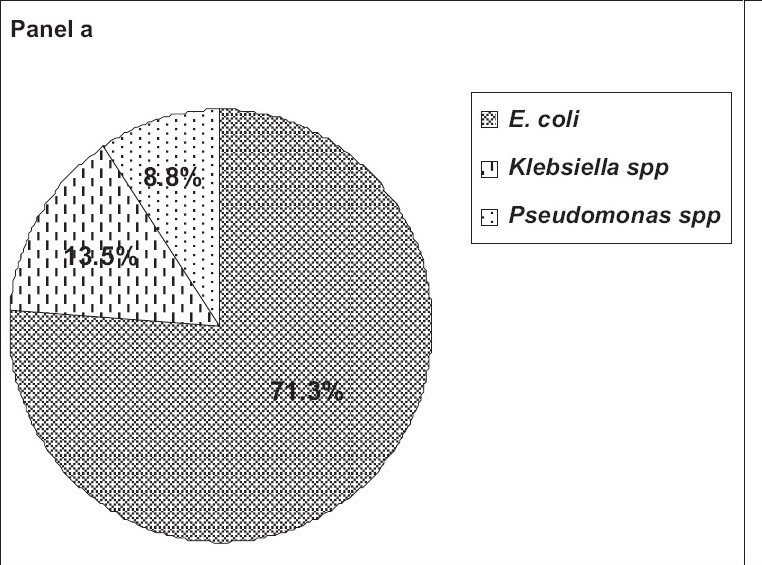
Panel a shows the percentage-wise distribution of gram negative bacilli among UTI subjects

Figure 1, panel b shows the percentage-wise distribution of gram positive cocci isolated from infected cases of both sexes. About 59% had *Enterococci* spp. followed by *coagulase-negative Staphylococcus* (25%). *Beta-hemolytic Streptococci* were isolated from 8% of the patients. *Nonhemolytic Streptococcus* was found in 6% and *Staphylococcus aureus* was seen in 2% of the patients.

**Figure 1:**
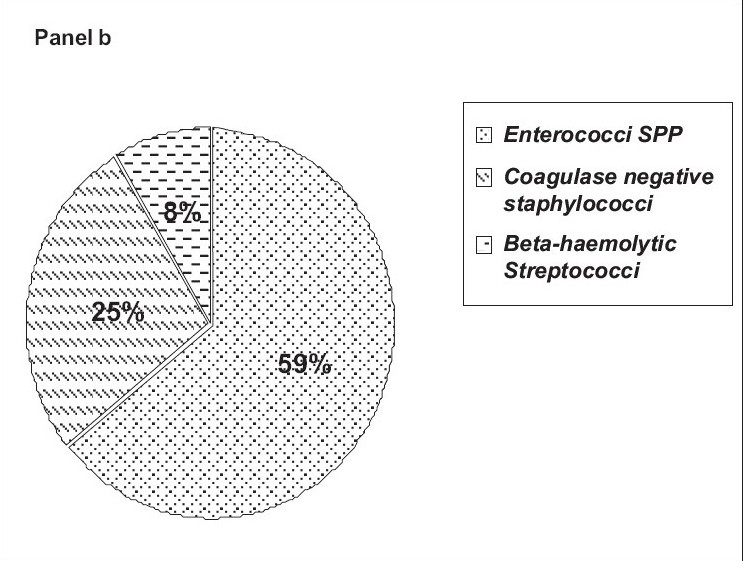
Panel b shows the percentage-wise distribution of gram positive cocci among UTI subjects

Among the specimens containing *Candida*, 57 (80.3%) were *Candida spp.* and 14 (19.7%) were *Candida albicans*.

Table 3 shows the antimicrobial pattern of both gram negative bacilli and gram positive cocci. Gram negative bacilli were found to be highly sensitive to sulbactum / cefoperazone (91%) and piperacillin / tazobactum (83%). Gram positive cocci were 75% sensitive to sulbactum / cefoperazone and 66% sensitive to piperacillin / tazobactum respectively.

**Table 3:** Antimicrobial pattern of gram positive cocci and gram negative bacilli

Antimicrobials	Gram positive cocci (*n* = 100) values are in percentages	Gram negative bacilli (*n* = 362)
Amikacin	29	65
Netillin	42	67
Tobramycin	14	30
Sulbactum / cefoperazone	75	91
Pipercillin / tazobactum	66	83
Ciprofloxacin	33	62
Ofloxacin	50	23
Norfoxacin	15	20
Cefoperazone	35	33
Ceftzoxime	48	62
Cefotaxime	63	51

Gram negative bacilli were found to be more sensitive than gram positive cocci to aminoglycosides such as netillin (67 *vs* 42%), amikacin (65 *vs* 29%), and Tobramycin (30 *vs* 14%).

Gram positive cocci (50%) were found to be more sensitive to ofloxacin than gram negative bacilli (23%) whereas gram negative bacilli (62%) were more sensitive than gram positive cocci (33%) to ciprofloxacin.

Not much difference in sensitivity was observed between gram positive cocci (35%) and gram negative bacilli (33%) to cefoperazone. Gram positive cocci (63%) were found to be more sensitive to cefotaxime than gram negative bacilli (51%), whereas gram negative bacilli (62%) were more sensitive than gram positive cocci (48%) to ceftizoxime.

## Discussion

In this study, we found that the prevalence of lower UTI was significantly higher in female than in male type 2 diabetic patients. Evidence from various epidemiological studies showed that UTI is more common in women with diabetes than those without diabetes.[13] The high level of infection in the urinary tract of diabetic women may be determined by the number of microorganisms located in the vagina.[14]

UTI appears to be multifactorial in subjects with diabetes and various diabetes-related risk factors have been proposed. We observed that age, longer duration of diabetes, and poor glycemic control were significantly associated with UTI among subjects with diabetes. The presence of UTI varied among nonobese and obese subjects. A study from the Netherlands showed that among women with diabetes, older age, proteinuria, a lower body mass index, and a history of UTI were important risk factors for UTI.[5]

In another study, the longer duration of diabetes but not glucose control, was associated with the prevalence of bacteriuria.[15] The association between glycemic control and UTI among diabetic patients is controversial. In our study, only a few cases showed recurrent urinary tract infection. Diabetes was found not to be a risk factor for recurrent urinary tract infections in postmenopausal women.[16]

Bacteriological studies usually reveal the involvement of gram negative enteric organisms that commonly cause urinary tract infections, such as *E. coli,* the *Klebsiella* species, and the *Proteus* species.[17] Similarly, the predominant number of pathogens isolated in our study were gram negative bacilli rather than gram positive pathogens.

Among the patients infected with gram negative bacilli in our study, *Escherichia coli* was isolated from 71% of the subjects, *Klebsiella* spp. from 13.5%, *Pseudomonas* spp. from 9%, *Enterobacter* spp. and *Citrobacter* spp. in 2%, and *nonfermenting gram negative bacilli* and *the Proteus* spp in 1%.

In another study from India, it was found that *E. coli* was the most commounly grown organism (64.3%), followed by *Staphylococcus aureus* (21.4%), and *Klebsiella pneumoniae* (14.3%).[18] In a recent study, it was noted that increased adherence of *E. coli* with type 1 fimbriae to uroepithelial cells isolated from the urine of women with diabetes correlated positively with HbA_*1C*._ Poorly controlled patients had a higher adherence of *E. coli.*[19]

Urinary tract infections due to *Enterococci* are quite common, particularly in patients who have received antibiotic treatment or who have undergone instrumentation of the urinary tract.[20] It has been reported that the prevalence of enterococci as a cause of nosocomial UTI increased between 1975 and 1984.[21] Lloyds *et al*. have shown that *Enterococcal* species accounted for 35% of urinary tract isolates.[22] Our results showed that 59% of subjects had *Enterococci spp.* among gram positive pathogens.

Gram positive cocci play a lesser role in UTIs. However, *Staphylococcs saprophyticus,* a novobiocin-resistant, coagulase-negative species, accounts for 10–15% of acute symptomatic UTIs in young females.[23] Therapy with antibiotics directed at the offending organisms is important while the underlying diabetes is effectively managed.[24] Due to the frequent (symptomatic) upper tract involvement and the possibly serious complications, many experts recommend a 7–14 day oral antibacterial cystitis treatment in these patients with an antibacterial agent that achieves high concentrations both in the urine and in the urinary tract tissue. There is a great likelihood that UTIs are affected by antimicrobial resistance or atypical uropathogens due to which the risk of upper tract involvement is increased.[25]

The choice of antibiotic therapy should integrate the local sensitivity pattern of the infecting organisms. For seriously ill patients including patients infected with *Pseudomonas*, agents such as Imipenem, Ticarcillin-clavulanate, and Piperacillin-tazobactum may also be considered.

Treatment of asymptomatic bacteriuria in patients with diabetes is often recommended to prevent the risk of symptomatic UTIs.[26]

We also found that sulbactum/cefoperazone and pipercillin/tazobactum were highly sensitive to both gram positive cooci and gram negative bacilli.

Gram negative bacilli were found to be more sensitive than gram positive cocci to aminoglycosides such as netillin, amikacin, and tobramycin. Netilmycin is a derivative of Gentamycin that is less nephrotoxic and ototoxic. It is less active against *Pseudomonas* but it inhibits a number of strains of *E. coli* as well as *Klebsiella* resistant to tobramycin.[27]

Gram negative bacilli were found to be highly sensitive to ciprofloxacin (62%) than to ofloxacin (23%). Ciprofloxacin is thus clearly useful against polyresistant species such as *Pseudomonas aeroginosa.*[28] Gram positive cocci (63%) were found to be more sensitive to cefotaxime than gram negative bacilli (51%). Ceftriaxone, ceftizoxime, and cefotaxime have excellent activity against *Streptococci.*[17] More than two urinary tract infections per year should alert physicians to possible cystpathy and should elicit appropriate diagnostic procedures.[29]

One of the limitations of this study was that a control group was not included for comparison. In summary, the prevalence of lower UTI was high in women with type 2 diabetes than in men. UTI was found to be associated with age, duration of diabetes, and poor glycemic control. *Escherichia coli* was commonly isolated; the gram negative pathogens were highly sensitive to sulbactum / cefoperazone and piperacillin / tazobactum.
